# Research on time-division multiplexing for error correction and privacy amplification in post-processing of quantum key distribution

**DOI:** 10.1038/s41598-024-77047-9

**Published:** 2024-10-25

**Authors:** Lei Chen, Xiao-Ming Chen, Ya-Long Yan

**Affiliations:** 1 School of Cyberspace Security, Beijing University of Posts and Telecommunications, No.10, Xitucheng Road, Haidian District, Beijing, 100876 Beijing China; 2 Department of Cyberspace Security, Beijing Electronic Science and Technology Institute, No.7, Fufeng Road, Fengtai District, Beijing, 100070 Beijing China; 3 School of Cyber Science and Technology, University of Science and Technology of China, No. 96, Jinzhai Road, Baohe District, Hefei, 230026 Anhui China

**Keywords:** Quantum key distribution, Error correction, Error-correcting codes, Privacy amplification, Universal hash function families, Quantum information, Information technology

## Abstract

The post-processing of quantum key distribution mainly includes error correction and privacy amplification. The error correction algorithms and privacy amplification methods used in the existing quantum key distribution are completely unrelated. Based on the principle of correspondence between error-correcting codes and hash function families, we proposed the idea of time-division multiplexing for error correction and privacy amplification for the first time. That is to say, through the common error correction algorithms and their corresponding hash function families or the common hash function families and their corresponding error-correcting codes, error correction and privacy amplification can be realized by time-division multiplexing with the same set of devices. In addition, we tested the idea from the perspective of error correction and privacy amplification, respectively. The analysis results show that the existing error correction algorithms and their corresponding hash function families or the common privacy amplification methods and their corresponding error-correcting codes cannot realize time-division multiplexing for error correction and privacy amplification temporarily. However, according to the principle of correspondence between error-correcting codes and hash function families, the idea of time-division multiplexing is possible. Moreover, the research on time-division multiplexing for error correction and privacy amplification has some practical significance. Once the idea of time-division multiplexing is realized, it will further reduce the calculation and storage cost of the post-processing process, reduce the deployment cost of quantum key distribution, and help to remote the practical engineering of quantum key distribution.

## Introduction

Quantum key distribution (QKD)^1^ is a technique that can provide the information-theoretic secure secret key for both parties (Alice and Bob) in long-distance communication. The security of the key is guaranteed by physical laws^2,3^, including the uncertainty principle and the single photon non-cloning theorem. In addition, the security of QKD-related theories has been proved strictly^4–7^. In recent years, QKD has made a lot of progress in theory and practice^8–14^.

QKD includes the quantum signal transmission process and post-processing process^15^. The former process is mainly to change the quantum states of Alice and Bob into approximately the same key, that is, the sifted key. However, the sifted key is not secure. The eavesdropper (Eve) may launch some kinds of attacks during the quantum transmission process to obtain some information about the sifted key without being discovered by Alice and Bob. The latter process mainly includes error correction and privacy amplification. Error correction is the adjustment of the approximately identical sifted key held by Alice and Bob to the exact same key. Privacy amplification is to compress the information obtained by Eve during the quantum transmission process and error correction process by some calculation or mapping method, which can make Alice and Bob get the shorter, identical, and completely secure key from the sifted key. In particular, the common calculation or mapping method used in privacy amplification is universal hash function families. At present, the error correction algorithms used in the post-processing are completely unrelated to the universal hash function families. However, the literature^16–18^ points out that there is a correspondence between error-correcting codes and hash function families. From the perspective of combining error correction and privacy amplification, they are in sequence. According to the corresponding relationship principle, if we can construct the hash function families corresponding to common error correction algorithms or the error-correcting codes corresponding to universal hash function families, then we can adopt the common error correction algorithms and their corresponding hash function families or the universal hash function families and their corresponding error-correcting codes to realize error correction and privacy amplification through the time-division multiplexing by the same device, which will greatly reduce the cost of calculation and storage in the post-processing process.

Based on the principle of correspondence between error-correcting codes and hash function families, we propose the idea of time-division multiplexing for error correction and privacy amplification for the first time. Firstly, we analyze the characteristics of hash function families corresponding to commonly used error correction algorithms in QKD, including BBBSS protocol, Cascade protocol, Winnow protocol, BCH code, Turbo code, LDPC code, Polar code, and find that they do not meet the requirements of the hash function family applicable to privacy amplification. Then, we construct the error-correcting codes corresponding to the universal hash function family (Toeplitz matrix) in privacy amplification and find that it cannot meet the requirements of error correction. In summary, time-division multiplexing for error correction and privacy amplification cannot be realized temporarily by using the common error correction algorithms and their corresponding hash function families or the common privacy amplification methods and their corresponding error-correcting codes in the existing QKD. However, according to the correspondence between error-correcting codes and hash function families, the idea of time-division multiplexing is possible. In addition, the research has some practical implications. Once the idea of time-division multiplexing for error correction and privacy amplification is realized, it will further reduce the deployment cost of the post-processing process, and help to promote the engineering and practical application of QKD.

The structure of this paper is organized as follows. In “Post-processing process”, we introduce the commonly used error correction algorithms and privacy amplification methods in the post-processing process of QKD. In “The analysis of time-division multiplexing for error correction and privacy amplification”, we first introduce the principle of correspondence between error-correcting codes and hash function families and then analyze the possibility of time-division multiplexing for common error correction algorithms and their corresponding hash function families from the perspective of error correction or common privacy amplification methods and their corresponding error-correcting codes from the perspective of privacy amplification, respectively. Finally, a conclusion is given in “Conclusion”.

## Post-processing process

The post-processing process mainly includes error correction and privacy amplification. In the following, we briefly introduce the error correction algorithms and privacy amplification methods commonly used in the existing post-processing process of QKD.

### Error correction

The error correction algorithms commonly used in QKD include two types, one is based on the binary search algorithm, and the other is based on the error-correcting codes. The former mainly includes BBBSS protocol^19^ and Cascade protocol^20^. The latter mainly includes Winnow protocol^21^, the error correction algorithms based on BCH code^22–24^, based on Turbo code^25^, based on LDPC code^26,27^ and based on Polar code^28,29^.

#### BBBSS protocol

In 1992, Bennett et al.^19^ proposed the BBBSS protocol, which is the earliest error correction algorithm in QKD. The core idea of BBBSS protocol is dichotomy and parity check. Its error correction process is roughly as follows:

1. Alice and Bob use the same distribution function to rearrange their sifted key to realize the randomization of error codes;
2. According to the estimated bit error rate, Alice and Bob group the sifted key. The length of a group should be as short as possible so that the number of bit errors in each group does not exceed one bit;
3. Alice and Bob perform parity checks on each of their groups and compare the checksums, respectively. If the parity of a corresponding group is the same, it indicates that there are no error codes in the group or there may be an even number of error codes. Otherwise, it indicates that there are an odd number of errors in the group. Then the group is divided into two subgroups, Alice and Bob calculate and compare the parity of each subgroup. This is done until the errors are found and corrected.

After the parity of each group is compared, the last bit of the group should be discarded in order to reduce information leakage. In addition, at the end of each round, the remaining key will be rearranged and grouped again. To keep the number of bit errors in each new group to less than one, we need to increase the size of the group, such as to twice the size of the original group. Repeat the process until all errors have been corrected. BBBSS protocol is simple to operate and easy to implement, but it requires a large number of interactions.

#### Cascade protocol

In 1993, Brassard et al.^20^ proposed the Cascade protocol with higher error correction efficiency based on BBBSS protocol. The difference between Cascade protocol and BBBSS protocol is that the location of each key is recorded before each round of error correction. After each round of error correction, the key bit is not discarded. Instead, according to the location of the detected error bit, the error group in the previous rounds of error correction is deduced back, and the smallest group is selected to continue error correction. This is because since the key bit is wrong, it proves that there are still an odd number of errors in the group that the key bit was previously in. In this way, Cascade protocol can correct error codes in a more timely and rapid manner, reduce the number of interactions, and correspondingly reduce the number of discarded key bits, thus improving error correction efficiency. To date, Cascade protocol is still widely used. However, Cascade protocol does not discard the key bit after each error correction and must record the location of the key, which increases the communication and storage overhead.

#### Winnow protocol

Since both BBBSS protocol and Cascade protocol are based on binary search, they must have more interactions. In order to solve the above problems, Buttler et al.^21^ first proposed the Winnow protocol based on error-correcting codes in 2003. The core idea of Winnow protocol is parity check and Hamming code, and its error correction process is as follows.

1. Alice and Bob also need to group their sifted key;
2. Alice and Bob calculate their respective parity sums and compare them. If the parity of the corresponding group is the same, the last bit of the group is discarded directly. Otherwise, discard the last bit of the group and then carry out Hamming error correction;
3. Hamming error correction process: Alice and Bob multiply the remaining key by the pre-shared check matrix to obtain their own syndrome, respectively. The position where the addition of syndrome (modulo two plus) is one is the position where the error code is located, and the error correction can be completed by directly reversing the error bit. Similarly, in order to ensure the security of the key, Alice and Bob need to discard the key bits that are the same as the syndrome bits after each Hamming error correction. Finally, Winnow protocol requires multiple rounds of error correction until all errors have been corrected.

Compared with BBBSS protocol and Cascade protocol, Winnow protocol uses Hamming error correction to reduce the number of interactions and improve the error correction rate. However, Winnow protocol needs to discard the key with the same number of syndrome after each Hamming error correction, resulting in low error correction efficiency. Meanwhile, since Winnow protocol adopts Hamming error correction, only one error can be corrected in each error correction process. When there is more than one error in a group, errors may increase instead of decrease after each error correction.

#### Based on BCH code

Since Winnow protocol can only correct one error at a time, the BCH code that can correct multiple errors at the same time is introduced into the error correction process of QKD^22–24^. The error correction algorithm based on BCH code is similar to Winnow protocol based on Hamming code. The only difference is that Hamming code is replaced by BCH code, and the whole error correction process is basically the same as Winnow protocol.

#### Based on Turbo code

In 2004, Nguyek et al.^25^ proposed to apply the Turbo code to the error correction process of QKD. The structure of Turbo code used in QKD is similar to that of Turbo code in traditional channels. In other words, convolutional encoder, interleaver, and deleter are needed at the encoding end (sender), and soft input and soft output decoder, interleaver, deleaver, and decision are needed at the decoding end (receiver). The difference is that in the encoding and decoding model of traditional channels, Alice needs to send the information bits and the encoded check bits together to Bob, and Bob uses the check bits to correct the errors of the received information. However, in the error correction process of QKD, Alice only needs to send the encoded check bits to Bob, who uses the received check bits to conduct iterative decoding for the sifted key in his hand, and then uses hash authentication to judge whether the decoding is successful. If it is successful, the decoding will be terminated. Otherwise, the iterative decoding will continue until the decoding is successful or the maximum number of iterations is reached.

#### Based on LDPC code

In 2004, Pearson^26^ proposed to apply the LDPC code to the error correction process of QKD. Its error correction process is roughly as follows.

1. According to the estimated bit error rate, Alice and Bob choose the appropriate shared check matrix;
2. Alice sends the eigenvector obtained by multiplying the sifted key with the check matrix to Bob;
3. Bob adopts the same method to calculate his own eigenvector and compares it with Alice’s eigenvector. If they are equal, it proves that the sifted key is the same for both parties. Otherwise, Bob combines his own sifted key, bit error rate, check matrix, eigenvector and Alice’s eigenvector, adopts the belief propagation (BP) algorithm to carry out iterative decoding until his eigenvector is the same as Alice’s eigenvector.

The﻿ error correction algorithm based on LDPC code only needs one interaction to complete error correction, which greatly reduces the number of interactions and improves the system throughput. On the other hand, the bit error rate changes constantly in the error correction, and the check matrix in the error correction algorithm based on LDPC code is very sensitive to the bit error rate. In order to achieve the optimization of error correction efficiency, the check matrix needs to be adjusted constantly according to the change of bit error rate. Thus, the communication parties need to store a large number of check matrices, which increases the storage overhead. In order to solve the above problems, Elkouss et al.^27^ proposed an adaptive error correction algorithm based on LDPC code.

#### Based on Polar code

In 2012, Jouguet et al.^28^ introduced the Polar code into QKD for the first time. The use of Polar code greatly improves the error correction rate and efficiency, especially in the case of long code. In 2014, Nakassis et al.^29^ presented multiple application modes of Polar code in the QKD environment and analyzed the number of keys consumed by the error correction process in each application mode. Compared with the error correction algorithm based on LDPC code, the error correction algorithm based on Polar code has the advantages of flexible rate change and lower coding complexity in QKD.

### Privacy amplification

After the error correction process, the keys of both Alice and Bob are identical. However, the key cannot be used as a secure key. Because during the quantum transmission process, Eve could launch some kinds of attacks to get some information about the key. Moreover, the error correction process will also give away some information. Therefore, the communication parties need to carry out the privacy amplification process to get the final secure key. Privacy amplification is the process of calculating or mapping a longer key to get a shorter but fully secure key. In privacy amplification, the most commonly used calculating or mapping method is to use the hash function families to hash the sifted key. It should be emphasized that not all hash function families can be used for privacy amplification. In particular, the hash function family suitable for privacy amplification in QKD generally needs to meet the following four requirements^30^:

1. The hash function family should be universal or nearly universal;
2. The number of bits to represent a particular hash function in the hash function family should be fairly low;
3. The hash function family should have a large input size and output size;
4. The computation of hash function in the hash function family should be efficient.

Here is a brief explanation of why these four requirements must be met. The first requirement is that universality directly affects the quality of generated key, that is, the more universal the hash function family, the better the security of the generated key. The second requirement stems from the fact that in a hash function family, the choice of the hash function is random, and such a choice needs to be passed between Alice and Bob. But it does not matter, because the choice of the hash function does not have to be secret. It is acceptable to represent a hash function with the number of bits required proportional to the input size. Here’s why we need both large input and large output. In QKD, Alice and Bob estimate the number of bits that need to be removed for privacy amplification in a statistical sense by comparing the sample key. Such estimation must use random and evenly distributed test samples over the QKD running blocks, or they may be misestimated due to time-dependent attacks. Increasing the number of test samples can improve the accuracy of statistical estimation, but on the other hand, it also reduces the number of samples available for key generation. The ideal is to have a large block size from which a large number of test samples can be extracted, but the proportion of the test samples in the block is small. As a trade-off, we want the input size of the hash function family to be as large as possible, and the output size to be correspondingly large. Finally, the computational efficiency of hash function has important practical significance. In the real-time application of QKD, the extraction of secure key should not take too much time. In addition, the rapid development of high-speed QKD systems requires that the processing speed of privacy amplification must be fast enough.

The following is a brief introduction to the definitions of hash function family and universal hash function family^17,18^.

#### Definition 1

A (*N*; *n*, *m*) hash function family is a set of $\mathcal {H}$ of *N* hash functions such that $h: \mathcal {X} \rightarrow \mathcal {Y}$ for each $h \in \mathcal {H}$, where $|\mathcal {X}|=n$ and $|\mathcal {Y}|=m$. Generally, $n \ge m$.

#### Definition 2

A (*N*; *n*, *m*)-hash function family, $\mathcal {H}$, is $\delta -universal$ provided that for any two distinct elements $x_1, x_2 \in \mathcal {X}$, there exist at most $\delta N$ hash functions $h \in \mathcal {H}$ such that $h(x_1)=h(x_2)$. The parameter $\delta$ is often referred to as the collision probability of the hash function family. We will use the notation $\delta -\textrm{U}$ as an abbreviation for $\delta -$ universal.

To more conveniently describe the universality of a hash function family to make it independent of the output size *m*, let $\varepsilon =\delta m$. Specifically, the hash function family is said to be universal if and only if $\varepsilon =1$; the hash function family is said to be approximately universal when $\varepsilon \approx 1$. In other words, the closer $\varepsilon$ is to 1, the more universal the hash function family becomes.

There are three kinds of universal hash function families commonly used to achieve privacy amplification, which are based on binary matrix^31–33^, modular arithmetic^30^, and finite field multiplication^34^.

First, let’s look at the first kind, the universal hash function family based on binary matrix^31^. Let $\mathcal {A}=\textrm{GF}(2)^n$, $\mathcal {B}=\textrm{GF}(2)^m$. For $\textbf{T}$, a $m \times n$ binary matrix, let $h_\textbf{T}(\textbf{x}) = \textbf{Tx}$ be the product of $\textbf{T}$ with the column vector $\textbf{x}$. Then, $\mathcal {H} =\{h_\textbf{T}: \textbf{T} \in \textrm{GF}(2)^{m \times n}\}$ is universal. In the above family, the identification of the hash function, namely the matrix $\textbf{T}$, requires *mn* bits, which is unfortunately not acceptable for the QKD application. For example, when the input length is $n \approx {10}^5$ and $m=O(n)$, then a number of bits of the order ${10}^{10}$ will need to be transmitted. Furthermore, the computation will be $O(mn)=O(n^2)$. Fortunately, we can limit the size of the hash function family by requiring that the matrix above be a Toeplitz matrix. This means that for any *i*, *j*, *t*, $\textbf{T}_{i, j} = \textbf{T}_{i+t, j+t}$ where $1 \le i, i+t \le m$, $1 \le j, j+t \le n$. Moreover, such hash function family $\mathcal {H}_{\textrm{Toeplitz}}$ is still universal^32,33^. The advantage of adopting Toeplitz matrix is that transferring a Toeplitz matrix of $m \times n$ requires at most $m+n-1$ bits, because Toeplitz matrix can be completely determined by its first row and first column. In addition, the multiplication of Toeplitz matrix can reduce the computational complexity from $O(n^2)$ to $O(n\log {n})$ by fast fourier transform (FFT). The concrete form of Toeplitz matrix is as follows:

1 $$\begin{aligned} \textbf{T} = \begin{bmatrix} t_{n} & t_{n-1} & t_{n-2} & t_{n-3} & \cdots & t_{3} & t_{2} & t_{1} \\ t_{n+1} & t_{n} & t_{n-1} & t_{n-2} & t_{n-3} & \ddots & t_{3} & t_{2} \\ \vdots & t_{n+1} & t_{n} & t_{n-1} & t_{n-2} & t_{n-3} & \ddots & t_{3} \\ t_{n+m-3} & \ddots & t_{n+1} & t_{n} & t_{n-1} & t_{n-2} & t_{n-3} & \vdots \\ t_{n+m-2} & t_{n+m-3} & \ddots & t_{n+1} & t_{n} & t_{n-1} & t_{n-2} & t_{n-3}\\ t_{n+m-1} & t_{n+m-2} & t_{n+m-3} & \cdots & t_{n+1} & t_{n} & t_{n-1} & t_{n-2} \end{bmatrix} \end{aligned}$$

The second kind is the universal hash function family based on modular arithmetic^30^, which has the following form:

2 $$\begin{aligned} \mathcal {H}_{n,m}=\left\{ h_{a,b}:a,b \in \mathbb {Z}_{2^n},\textrm{gcd} \left( a,2\right) =1,b \ne 0 \right\} , \end{aligned}$$

where $h_{a,b}(x)=\lfloor (ax+b~\textrm{mod}~2^n)/2^{n-m}\rfloor$, $x \in \mathbb {Z}_{2^n}$. Compared with other types of universal hash function families, the computational efficiency of the universal hash function family based on modular arithmetic is relatively high. However, fully describing such a hash function in this family requires 2*n* bits, which is more than the number of bits needed to describe a Toeplitz matrix. In general, $n > m$^15^.

The third is the universal hash function family based on finite field multiplication^34^. Let $\mathcal {A}=\textrm{GF}(2^n)$, $\mathcal {B}=\left\{ 0,1\right\} ^m$. Let $h_c\left( x\right)$ be defined as the first *m* bits of the product *cx* in a polynomial representation of $\textrm{GF}\left( 2^n\right)$. The set

3 $$\begin{aligned} \mathcal {H}_{\textrm{GF}\left( 2^n\right) \rightarrow \left\{ 0,1\right\} ^m}=\left\{ h_c:c\in \textrm{GF} \left( 2^n\right) \right\} \end{aligned}$$

is a universal hash function family. Specially, the locations of the *m* extracted bits do not matter for the family to be universal. In addition, describing one of the hash functions requires only *n* bits (that is, the value of *c*).

## The analysis of time-division multiplexing for error correction and privacy amplification

### The correspondence between error-correcting codes and hash function families

In order to better explain the correspondence between error-correcting codes and hash function families, we briefly introduce the error-correcting codes and array representation of hash function families.

Let $\mathcal {Y}$ be an alphabet of *q* symbols. An $\left( N, K, d, q\right)$ code is a set $\mathcal {C}$ of *K* vectors (called codewords) in $\mathcal {Y}^N$ such that the Hamming distance between any two distinct vectors in $\mathcal {C}$ is at least *d*. If the code is linear (i.e., if *q* is a prime power, $\mathcal {Y}=\mathbb {F}_q$, and $\mathcal {C}$ is a subspace of $\left( \mathbb {F}_q\right) ^N$), then we will say that the code is an $\left[ N,k,d,q\right]$ code, where $k=\log _q{K}$ is the dimension of the code^17,18^.

We will often depict a $\left( N;n,m\right)$ hash function family, say $\mathcal {H}$, in the form of a $N\times n$ array of *m* symbols, where the rows are indexed by the functions in $\mathcal {H}$, the columns are indexed by the elements in $\mathcal {X}$, and the entry in row *h* and column *x* of the array is $h\left( x\right)$ (for every $h\in \mathcal {H}$ and every $x\in \mathcal {X}$). Each row of the array corresponds to one of the functions in the family. We denote this array by ${Array}(\mathcal {H})$ and call it an array representation of the hash function family $\mathcal {H}$. If $\mathcal {H}$ is a $\delta - \mathrm{{U}}(N;n,m)$ hash function family, then in any two columns of ${Array}(\mathcal {H})$, it follows that there exist at most $\delta N$ rows of ${Array}\left( \mathcal {H}\right)$ such that the entries in the two given columns are equal^17,18^.

It was Bierbrauer et al.^16^ who first pointed out the correspondence between error-correcting codes and hash function families. D.R. Stinson later gave it in the form of the following theorem^17,18^.

#### Theorem 1

*If there exists an*$\left( N,K,d,q\right)$*code, then there exists a*$\left( 1-\frac{d}{N}\right) -\mathrm{{U}}(N;K,q)$*hash function family*. *Conversely, if there exists an*$\delta - \mathrm{{U}}(N;n,m)$*hash function family, then there exists an*$(N,n,N\left( 1-\delta \right) ,m)$*code*.

#### Proof

Suppose $\mathcal {C} = \{C_1, \dots , C_K \}$ is the hypothesized code. Construct an $N \times K$ array, *Array*, in which the columns are the codewords in $\mathcal {C}$. If we look at any two columns of *Array*, we see that they contain different entries in at least *d*. Setting $d = (1-\delta )N$, the associated hash function family has $\delta = 1-d/N$.

The process can be reversed: by taking the columns of the array associated with an $\delta - \mathrm{{U}}(N;n,m)$ hash function family as codewords of a code, we obtain an $\left( N, K, d, q\right)$ code with $d \ge N(1-\delta )$. $\square$

Theorem 1 states that there is a correspondence between error-correcting codes and hash function families. Specifically, let the codewords in the stated codes correspond to the columns of ${Array}(\mathcal {H})$, where $\mathcal {H}$ is the stated hash function family. A specific example is given below^17^. The array representation of the hash function family is shown in Table 1.

#### Example 1

The following $\frac{1}{3} - \mathrm{{U}}(3;9,3)$ hash function family $\left\{ h_i:i\in \mathbb {Z}_3\right\}$ is equivalent to a $\left( 3,9,2,3\right)$ code.

**Table 1:** Array representation of the hash function family $\frac{1}{3} - \mathrm{{U}}(3;9,3)$.

	(0, 0)	(0, 1)	(0, 2)	(1, 0)	(1, 1)	(1, 2)	(2, 0)	(2, 1)	(2, 2)
$h_0$	0	0	0	1	1	1	2	2	2
$h_1$	0	1	2	1	2	0	2	0	1
$h_2$	0	2	1	1	0	2	2	1	0

As is shown in Table 1, there are three hash functions in the above family, namely $h_0$, $h_1$, and $h_2$. There are nine possible inputs to the hash function. When you use a ternary field, you need two bits to represent the input, They are $\left( 0,0\right)$, $\left( 0,1\right)$, $\left( 0,2\right)$, $\left( 1,1\right)$, $\left( 1,1\right)$, $\left( 2,0\right)$, $\left( 2,1\right)$ and $\left( 2,2\right)$. There are three possible outputs of the hash function. When a ternary field is used, a bit is needed to represent the output, namely 0, 1, 2. Taking input $\left( 0,0\right)$ as an example, when the hash function $h_0$ is selected, its output is 0, that is, $h_0\left( 0,0\right) =0$. Accordingly, the codeword is expressed in the ternary system. The code length of the code block is 3, the number of codewords is 9, and the Hamming distance is 2. Each column of ${Array}\left( \mathcal {H}\right)$ is a codeword. For example, the codeword for the first column is $\left( 0,0,0\right) ^\textrm{T}$.

### From the perspective of error correction

This part will start from the perspective of error correction, based on the commonly used error correction algorithms in QKD and Theorem 1, and construct the hash function families corresponding to the common error correction algorithms. Then, by analyzing whether the constructed hash function families meet the requirements of the hash function family applicable to privacy amplification, we will judge whether the common error correction algorithms and their corresponding hash function families can realize time-division multiplexing for error correction and privacy amplification.

#### Based on BBBSS protocol or Cascade protocol

According to Section 2.1, in the BBBSS protocol and Cascade protocol, Alice and Bob only continuously compare their parity sums to locate and correct error codes, but do not involve error-correcting codes. Therefore, we cannot make use of the correspondence between error-correcting codes and hash function families, cannot find the hash function families corresponding to BBBSS protocol and Cascade protocol through Theorem 1, and also cannot realize time-division multiplexing for error correction and privacy amplification.

#### Based on Winnow protocol

Winnow protocol is an error correction algorithm based on Hamming code. According to Theorem 1, if there exists a Hamming code $\left[ N,k,d,q\right]$, where the code length is $N=2^r-1$, the number of information bits is $k=2^r-1-r$, the minimum Hamming distance is $d=3$, the base number is $q=2$, the number of check bits is $r\ge 3$ and is an integer, then there must be a hash function family,

$$\begin{aligned} \left( 1-\frac{3}{2^r-1}\right) -\mathrm{{U}}(2r-1, 2r-1-r, 2). \end{aligned}$$

At this point,

4 $$\begin{aligned} & \delta =1-\frac{3}{2^r-1}, \end{aligned}$$

5 $$\begin{aligned} & \varepsilon =\delta q=2-\frac{6}{2^r-1}. \end{aligned}$$

Obviously, $\varepsilon$ is an increasing function of *r*, so we have

6 $$\begin{aligned} \varepsilon \ge \frac{8}{7}>1, \left( r\ge 3\right) . \end{aligned}$$

That is, the hash function family constructed by the Hamming code does not satisfy the first requirement for the hash function family applicable in privacy amplification—universal $\left( \varepsilon =1\right)$ or approximately universal $\left( \varepsilon \approx 1\right)$. In addition, Winnow protocol generally uses $\left( 7,4\right)$ Hamming code first. In this case, the input and output sizes of the corresponding approximate universal hash function family are both small, in which the number of input bits is 4 and the number of output bits is 1. Obviously, this does not satisfy the third requirement for a hash function family that applies to privacy amplification. If we want to increase the number of input bits, we have to increase *r* and $\varepsilon$, which will cause the corresponding hash function family to deviate even more from universality. Moreover, the Hamming code is defined in the binary field, and the number of output bits can only be 1.

Therefore, it is impossible to realize time-division multiplexing for error correction and privacy amplification by Winnow protocol based on Hamming code and its corresponding hash function family.

#### Based on BCH code

Similarly, the basic BCH code is also defined in the binary field, and the output of the corresponding hash function family is only one bit. The number of output bits is too small, which also fails to meet the third requirement for the hash function family in the privacy amplification, that is, large input size and large output size.

Therefore, the error correction algorithm based on BCH code cannot be combined with its corresponding hash function family to realize time-division multiplexing for error correction and privacy amplification.

In particular, although the multi-base BCH code, also known as Reed-Solomon (RS) code^35^, has not been used in the error correction of QKD, as a special BCH code, it is briefly analyzed here. On the one hand, RS code adopts multi-digit encoding rather than binary encoding, and has more output bits of the corresponding hash function compared with the basic BCH code, so it may be able to meet the third requirement of the hash function family applicable to privacy amplification. On the other hand, the length of RS code is odd. In order to obtain codewords with even lengths and increase the ability of error detection, people generally use extended RS code. The extended RS code is no longer cyclic, the decoding process is more complex, and the computational efficiency of the corresponding hash function family is lower^36^. Therefore, it cannot meet the fourth requirement of the hash function family applicable to privacy amplification.

In summary, under the existing conditions, the error correction algorithm based on RS code and its corresponding hash function family cannot realize the time-division multiplexing for error correction and privacy amplification.

#### Based on Turbo code or LDPC code or Polar code

For Turbo code, because they are no longer linear codes, the calculation of the minimum Hamming distance is relatively complicated at present^37^. For LDPC code, the exact calculation of its minimum Hamming distance is a non-deterministic polynomial complete (NPC) problem. In other words, the time complexity of calculating the minimum Hamming distance of LDPC code cannot be expressed by a polynomial^38–41^. As for Polar code, it does not consider the minimum distance characteristic at the beginning of its design, but uses channel combination and channel splitting to choose a specific coding scheme. Moreover, a probabilistic algorithm is used in decoding^42^. In general, the minimum Hamming distance *d* for the error correction algorithms based on Turbo code or LDPC code or Polar code in post-processing cannot be calculated quantitatively and accurately, which results in the first requirement for a hash function family applicable to privacy amplification—universality cannot be measured well.

In addition, for the convenience of representation and calculation, Turbo code, LDPC code and Polar code all adopt binary coding. Therefore, like Winnow protocol based on Hamming code and the error correction algorithm based on BCH code, the number of output bits of hash function family constructed based on Turbo code, LDPC code or Polar code is too small, only one bit. They do not meet the third requirement for a hash function family applicable to private amplification—large input and output sizes.

Based on the above two reasons, the time-division multiplexing for error correction algorithms based on Turbo code, LDPC code or Polar code and their corresponding hash function families cannot be realized at present.

The hash function families, which are directly converted by the common error correction algorithms in the post-processing of QKD through Theorem 1, are compared and summarized with the requirements for the hash function family suitable for privacy amplification, as shown in Table 2. In Table 2, “$\checkmark$” indicates that the requirement is met, “$\times$” indicates that the requirement is not met, and “-” indicates that the requirement is not analyzed. Because all requirements need to be met before the hash function family can be used for privacy amplification. It can be seen from Table 2 that the hash function families corresponding to common error correction algorithms cannot simultaneously meet the requirements for the hash function family applicable to privacy amplification. That is to say, from the perspective of error correction, the existing error correction algorithms commonly used in the post-processing of QKD and their corresponding hash function families according to Theorem 1 cannot realize time-division multiplexing for error correction and privacy amplification temporarily.

**Table 2:** According to Theorem 1, the hash function families transformed by the common error correction algorithms are compared with the requirements of the hash function family in privacy amplification.

The bases				
Reqs	Req 1	Req 2	Req 3	Req 4
BBBSS protocol or Cascade protocol	$\times$	$\times$	$\times$	$\times$
Winnow protocol	$\times$	-	$\times$	-
BCH code	-	-	$\times$	-
RS code	-	-	$\checkmark$	$\times$
Turbo code or LDPC code or Polar code	$\times$	-	$\times$	-

### From the perspective of privacy amplification

The Toeplitz matrix is the most commonly used universal hash function family in the privacy amplification of QKD. Therefore, in this part, we will construct the error-correcting codes based on Toeplitz matrix and Theorem 1 from the perspective of privacy amplification. Then, by analyzing whether the constructed error-correcting codes meet the requirements of the error correction algorithm in the error correction of QKD, we can judge whether the corresponding error-correcting codes and Toeplitz matrix can realize time-division multiplexing for error correction and privacy amplification.

Suppose that the Toeplitz matrix $\textbf{T}_{m\times n}$ and key column vector $\textbf{S}_{n\times 1}$ with *m* rows and *n* columns, then the key column vector after hashing $\textbf{R}_{m\times 1}=\textbf{T}_{m\times n}\cdot \textbf{S}_{n\times 1}$, that is,

7 $$\begin{aligned} \begin{bmatrix} r_{1} \\ r_{2}\\ \vdots \\ r_{m-2}\\ r_{m-1} \\ r_{m} \end{bmatrix} = \begin{bmatrix} t_{n} & t_{n-1} & t_{n-2} & t_{n-3} & \cdots & t_{3} & t_{2} & t_{1} \\ t_{n+1} & t_{n} & t_{n-1} & t_{n-2} & t_{n-3} & \ddots & t_{3} & t_{2} \\ \vdots & t_{n+1} & t_{n} & t_{n-1} & t_{n-2} & t_{n-3} & \ddots & t_{3} \\ t_{n+m-3} & \ddots & t_{n+1} & t_{n} & t_{n-1} & t_{n-2} & t_{n-3} & \vdots \\ t_{n+m-2} & t_{n+m-3} & \ddots & t_{n+1} & t_{n} & t_{n-1} & t_{n-2} & t_{n-3}\\ t_{n+m-1} & t_{n+m-2} & t_{n+m-3} & \cdots & t_{n+1} & t_{n} & t_{n-1} & t_{n-2} \end{bmatrix} \begin{bmatrix} s_{1} \\ s_{2}\\ \vdots \\ s_{n-2}\\ s_{n-1} \\ s_{n} \end{bmatrix} \end{aligned}$$

The above Toeplitz matrix is represented in the form of $\delta - \textrm{U}$, namely

$$\begin{aligned} \frac{1}{2^m}-\mathrm{{U}}(2^{n+m-1};2^n,2^m). \end{aligned}$$

To simplify the analysis, we directly adopt $\varepsilon =1$. According to the description of Theorem 1, the error-correcting codes corresponding to this hash function family can be expressed as

$$\begin{aligned} \left( 2^{n+m-1}, 2^n, 2^{n+m-1}-2^{n-1}, 2^m\right) . \end{aligned}$$

The error correction performance of this code is analyzed below, as shown in Table 3. On the one hand, the Hamming distance of the error-correcting codes is $d=2^{n-1}(2^m -1)$, and it is large, so the error-correcting ability is relatively strong. But on the other hand, the code length is $2^{n+m-1}$, the number of information bits is $\mathrm{{log}}_{2^m}^{2^n} = \frac{n}{m}$, and the code rate is

8 $$\begin{aligned} rate=\frac{n}{m\cdot 2^{n+m-1}} \ll 1. \end{aligned}$$

Therefore, the code rate is too low. In addition, according to literature^43^, when error-correcting codes are used in the error correction process of QKD, the information leakage is approximately the number of check bits, that is,

9 $$\begin{aligned} {leak}_{\textrm{EC}}\approx 2^{n+m-1}- \frac{n}{m}. \end{aligned}$$

According to formula (9), it can be seen that the error-correcting codes leak too much information, and the error correction efficiency is too low.

**Table 3:** The performance of Toeplitz matrix corresponds to the error-correcting codes.

Performance Index	Index Value	Conclusion
Hamming distance	$d = 2^{n-1}(2^m -1)$	Relatively strong
Code rate	$rate=\frac{n}{m\cdot 2^{n+m-1}} \ll 1$	Too low
Information leakage	${leak}_{\textrm{EC}}\approx 2^{n+m-1}- \frac{n}{m}$	Too much

In summary, although the error-correcting code corresponding to the Toeplitz matrix has a strong error-correcting ability, its code rate is too low, the information leakage is too large, and the error correction efficiency is too low. Thus, it still cannot be used in the error correction of QKD. In other words, from the perspective of privacy amplification, the existing privacy amplification method (i.e., Toeplitz matrix) commonly used in QKD and its error-correcting codes constructed through Theorem 1 cannot achieve time-division multiplexing for error correction and privacy amplification.

## Conclusion

Based on the principle of correspondence between error-correcting codes and hash function families, the idea of time-division multiplexing for error correction and privacy amplification is proposed for the first time in order to reduce the calculation and storage overhead of the post-processing in QKD. The analysis results show that the hash function families corresponding to the common error correction algorithms in QKD constructed based on the correspondence relation principle cannot meet the requirements for the hash function family applicable to the privacy amplification process, and the error-correcting codes corresponding to the common privacy amplification methods in QKD constructed based on the correspondence relation principle cannot meet the requirements for the error-correcting codes applicable to the error correction process. Therefore, the common error correction algorithms and their corresponding hash function families or the common privacy amplification methods and their corresponding error-correcting codes in the existing QKD cannot realize time-division multiplexing for error correction and privacy amplification temporarily.

However, the present research results do not show that the idea of time-division multiplexing for error correction and privacy amplification is not feasible. On the contrary, it shows that more research is needed. As a cryptography and coding expert D.R. Stinson put it in his paper^18^, “We have also emphasized the close connections between universal hash families, error-correcting codes and orthogonal arrays. $\cdots$ It is our belief that coding theory is the ‘right’ way to view hash families and that the enormous amount of research on coding theory in the last 50 or so years has not been exploited to its full potential in the study of universal hash families and their many applications”. At the same time, relevant studies show that the integration of sifting, error correction and privacy amplification into one process can increase quadratically the size of the secret key allowing to raise up the secret key rate at least theoretically, such as the method of binary frames^44^. In addition, the research on time-division multiplexing for error correction and privacy amplification has some practical significance. Once the idea of time-division multiplexing is realized, it will further reduce the calculation and storage cost of post-processing, reduce the equipment deployment cost of QKD, and help to promote the engineering and practical application of QKD.

## Data Availability

All data generated or analysed during this study are included in this published article.
